# An Unexpected Recovery

**Published:** 1898-07

**Authors:** 

**Affiliations:** Veterinary Surgeon, Ottawa, Canada; Veterinary Surgeon, Ottawa, Canada


### AN UNEXPECTED RECOVERY.

By James and Perley, Veterinary Surgeons,
OTTAWA, CANADA.

On July 5, 1898, at 10 A.M., we were summoned to attend a case of colic.

*Subject:* A seven-year-old draught-horse, weighing about 1400 pounds.

*History:* Animal was taken sick about half an hour previous to seeing him, and while at work. When first seen he presented all the symptoms of spasmodic colic, and was treated for such without any apparent relief.

About one o'clock tympanites was quite marked, and we were almost on the point of using a trocar, when the distention rapidly disappeared. At this stage the animal began sitting on his haunches and balancing himself on his back. There had been no movement of the bowels up to this time; pulse weak and about 80 beats per minute; temperature normal.

We now diagnosed case as one of volvulus or intussusception, with prognosis very unfavorable, and administered two grains each of eserine and pilocarpine hypodermically, and in ten minutes the animal began to strain, but passed nothing. In twenty minutes more animal was very uneasy, and there occurred a regurgitation of a clear liquid amounting to about a pint.

During the next hour about three-quarts of ingesta were vomited and animal became very weak; temperature rose to 103° F., and pulse became almost obliterated. Injections were used without avail, and a weak mustard-plaster applied to abdomen. Catheter was passed and a considerable quantity of normal urine drawn off.

At 6 P. M. the animal appeared better, and from that time improved rapidly. He was given light diet the following day, and is at his usual work to-day as well as ever.

